# Reduced Quantitative Ultrasound Bone Mineral Density in HIV-Infected Patients on Antiretroviral Therapy in Senegal

**DOI:** 10.1371/journal.pone.0031726

**Published:** 2012-02-16

**Authors:** Amandine Cournil, Sabrina Eymard-Duvernay, Assane Diouf, Claire Moquet, Julie Coutherut, Ndèye Fatou Ngom Gueye, Cécile Cames, Bernard Taverne, Kirsten Bork, Papa Salif Sow, Eric Delaporte, for the ANRS 1215 Study Group

**Affiliations:** 1 Institut de Recherche pour le Développement/University Montpellier 1, UMI 233, Montpellier, France; 2 Regional Research and Training Centre for HIV/AIDS, Fann University Teaching Hospital, Dakar, Senegal; 3 Institut de Recherche pour le Développement/University Montpellier 1, UMI 233, Dakar, Senegal; 4 Ambulatory Care Unit, Fann University Teaching Hospital, Dakar, Senegal; 5 Department of Infectious Diseases, Fann University Teaching Hospital, Dakar, Senegal; University of New South Wales, Australia

## Abstract

**Background:**

Bone status in HIV-infected patients on antiretroviral treatment (ART) is poorly documented in resource-limited settings. We compared bone mineral density between HIV-infected patients and control subjects from Dakar, Senegal.

**Methods:**

A total of 207 (134 women and 73 men) HIV-infected patients from an observational cohort in Dakar (ANRS 1215) and 207 age- and sex-matched controls from the general population were enrolled. Bone mineral density was assessed by quantitative ultrasound (QUS) at the calcaneus, an alternative to the reference method (i.e. dual X-absorptiometry), often not available in resource-limited countries.

**Results:**

Mean age was 47.0 (±8.5) years. Patients had received ART for a median duration of 8.8 years; 45% received a protease inhibitor and 27% tenofovir; 84% had undetectable viral load. Patients had lower body mass index (BMI) than controls (23 versus 26 kg/m^2^, P<0.001). In unadjusted analysis, QUS bone mineral density was lower in HIV-infected patients than in controls (difference: −0.36 standard deviation, 95% confidence interval (CI): −0.59;−0.12, P = 0.003). Adjusting for BMI, physical activity, smoking and calcium intake attenuated the difference (−0.27, CI: −0.53;−0.002, P = 0.05). Differences in BMI between patients and controls explained a third of the difference in QUS bone mineral density. Among patients, BMI was independently associated with QUS bone mineral density (P<0.001). An association between undetectable viral load and QUS bone density was also suggested (β = 0.48, CI: 0.02;0.93; P = 0.04). No association between protease inhibitor or tenofovir use and QUS bone mineral density was found.

**Conclusion:**

Senegalese HIV-infected patients had reduced QUS bone mineral density in comparison with control subjects, in part related to their lower BMI. Further investigation is needed to clarify the clinical significance of these observations.

## Introduction

Reduced bone mineral density is a recognized metabolic complication of HIV and its treatment. The prevalence of osteoporosis is estimated to be around threefold-higher in HIV-infected individuals than in uninfected controls [1], [2]. Several studies indicate that HIV infection is also associated with increased though modest risk of fracture [3], [4]. Despite growing concern about this complication, the impact on bones of HIV and its treatment in resource-limited countries is poorly documented. To our knowledge, no study has been conducted on this subject in Sub-Saharan Africa, although this region houses two-thirds of the persons living with HIV worldwide. This situation is primarily due to the lack of specific equipment for assessing bone mineral density by dual-energy x-ray absorptiometry (DXA). DXA is the standard reference method for diagnosing osteoporosis, but requires expensive equipment not currently available in most resource-constrained regions. Meanwhile, recent developments in densitometry technology have provided alternative methods, among which heel quantitative ultrasound (QUS) appears to be the most widely used [5]. It is inexpensive, portable, ionizing-radiation-free and proven to predict hip fractures and all osteoporotic fractures in Caucasian postmenopausal women and elderly men [6], [7], [8], [9]. Although its use in clinical practice is still not well defined and depends on the type of device; for epidemiological purposes, it provides an appropriate tool for comparing bone mineral density between different groups and identifying factors associated with variation in bone density, especially in settings where DXA is not available [10], [11].

The first objective of this study was to compare QUS bone density between HIV-infected patients initially included in an observational cohort (ANRS 1215) in the city of Dakar, Senegal, and a group of age- and sex-matched control subjects from the general population of Dakar. A second objective was to assess the effect of traditional and HIV-related factors on QUS bone mineral density in HIV-infected patients.

## Methods

### Study design

A cross-sectional exposed/unexposed study was conducted in Dakar, Senegal in 2010. Each HIV-infected patient (exposed) was matched for sex, age (+/−5 years) and living area to a control subject (unexposed) from the general population.

### Study population

HIV-infected patients were recruited within the ANRS 1215 observational cohort which initially included 404 HIV-1 patients enrolled in the Senegalese antiretroviral drug access initiative between 1998 and 2002 [12], [13], [14]. Forty patients enrolled in a subsequent clinical trial in 2004 were later pooled into the ANRS 1215 cohort [15]. All patients were receiving antiretroviral treatment (ART). The initial ART regimen was a triple drug combination: two nucleoside reverse transcriptase inhibitors (NRTI) + a non-nucleoside reverse transcriptase inhibitor (NNRTI) or a protease inhibitor (PI), except for 18 patients who received only two NRTIs.

### Participants

Among the 444 patients included in the ANRS 1215 cohort, 125 had died and 52 were lost-to-follow-up. Finally, a total of 267 patients who were still alive and followed on June, 30^th^, 2010 at the Fann University Hospital in Dakar were eligible.

Patients were recruited between April and June 2010 during clinical follow-up visits. Among 231 patients who presented for routine examination and were invited to join, 218 agreed to participate and were enrolled. Patient clinical and biological characteristics included date of ART initiation, AIDS stage according to US Centers for Disease Control and Prevention (CDC) classification, hepatitis C virus serological status at inclusion, type and duration of all antiretroviral treatments received since initiation, initial and current CD4 cell count and viral load, nadir CD4 cell count and body mass index (BMI) at inclusion in the cohort. All these variables were extracted from the ANRS 1215 cohort data base.

Control subjects were recruited at home in 9 districts of the city of Dakar and its outskirts, defined according to the place of residence of the patients. In each district, controls were recruited randomly as long as they could be sex- and age-matched to a patient from this area. Patients from outside Dakar or its surrounding were randomly assigned to one of the 9 districts for the purpose of control selection. The response rate for recruitment of control subjects was 54%.

Considering the low prevalence of HIV in Dakar and its surroundings (<1%), controls were presumed to be HIV-negative [16].

### Clinical evaluation

Data documenting risk factors for low bone mineral density were collected during a face-to-face interview for both patients and controls. They included previous fracture, prior fracture in a first-degree relative, smoking status, alcohol consumption, corticosteroid use, medical history of specific pathologies (rheumatoid arthritis, secondary osteoporosis) and menopausal status. In patients, medical information was validated by clinicians. Calcium intake was assessed by a 7-day semi-quantitative food frequency questionnaire that included 17 calcium-rich food items commonly consumed in Dakar [17]. Physical activity was assessed by a short frequency questionnaire (‘regular physical activity’ was defined as moderate activity each day and strenuous activity at least once a month).

Weight and height were measured during the interview to calculate BMI = weight (kg)/height(m)^2^.

### QUS bone mineral density

Bone mineral density was assessed using a heel ultrasonic gel-coupled QUS system, the Pegasus Prestige ultrasonometer (Osteomed, DMS, France). The Pegasus Prestige ultrasound device measures two parameters at mid-calcaneus: bone ultrasound attenuation (BUA) (in dB/MHz) and speed of sound (in m/s). Three repeated measurements with repositioning were performed on the same foot for all participants. The in vivo coefficient of variation for BUA was 3.3% in patients and 3.7% in controls.

BUA was then expressed as T-score (standard deviations from the mean value in normal young individuals of the same sex) or Z-score (standard deviations from the mean in individuals of the same age and sex) using the manufacturer's age- and sex-specific reference data for a Caucasian population.

### Ethics statement

The Senegalese ethics committee, or Comité National d'Éthique pour la Recherche en Santé (CNERS), Direction de la Santé, Ministère de la Santé et de la Prévention, Dakar, Sénégal, approved the study and all participants gave informed consent. Authorizations from the committee were obtained separately for the cohort (patients) in April 2009 and for controls in September 2010. Patients signed an informed consent form at the time of inclusion in the cohort. Control participants signed an informed consent form when the survey was conducted in 2010.

### Statistical analysis

Comparison of patients included and not included in the study were made using Student's t-test for normally distributed continuous variables and Mann-Whitney-U test for continuous variables not normally distributed, using the χ^2^-test for categorical variables.

Comparison of characteristics in patients and control subjects were made using logistic and linear models. We accounted for the matched-pairs design by introducing the pair-ID as a fixed effect in the models.

To assess whether T-score (outcome variable for bone density assessment) differed between patients and controls, we built a first multivariate linear model with the T-score being the dependent variable; independent variables were those that met the criterion of P<0.20 in bivariate analysis for association with the T-score (i.e. BMI and physical activity). We then built a second model which included, in addition, variables not associated with T-score (at a P-value threshold of 0.20), but which differed between patients and controls (i.e. calcium intake and smoking). A sex-related effect modification was investigated through interaction terms in the multivariate model.

To identify factors associated with T-score among patients, we built a multivariate model including variables that met the criterion of P<0.20 in bivariate analysis for association with the T-score. Since only 3 patients reported alcohol consumption and none reported rheumatoid arthritis, secondary osteoporosis or exposure to glucocorticoids, these risk factors for low bone mineral density or osteoporosis were not presented or included in analyses.

All statistical analyses were performed using Stata software (version 10.1, Stata Corp, College Station, TX, USA). Tests were considered statistically significant for P<0.05.

## Results

### Clinical characteristics of HIV-infected patients

218 out of 267 eligible patients were enrolled in the present study and 207 patients were finally included in the analysis. Reasons for non-inclusion were pregnancy (n = 5) or missing data for the patient him/herself or his/her control counterpart (n = 6). Compared with patients included in the analysis, non-enrolled or non-analyzed patients were more likely to be male (58.3% vs 35.3%; P = 0.001) and to have received PI (60.0% vs 44.9%; P = 0.04) or tenofovir disoproxyl fumarate (TDF) (43.3% vs. 26.6%; P = 0.01).

Seventy-five percent of patients had been receiving antiretroviral treatment for at least 8 years (Table 1). At the time of the study, 70% were receiving two NRTIs and one NNRTI; 20% received two NRTIs in association with a PI and 10% were on bitherapy (TDF and one NNRTI). The current median HIV viral load was 1.7 (range: 1.6;6.7) log copies/mL and 84% of patients were undetectable.

**Table 1:** QUS bone mineral density and lifestyle factors according to HIV status and clinical characteristics of HIV-infected patients.

	HIV+ patientsn = 207		Controlsn = 207		P
Bone mineral density					
BUA, mean ± SD dB/MHz	64.0	±7.0	66.0	±7.9	0.003
T-score, mean ± SD	−0.70	±1.23	−0.34	±1.39	0.003
Z-score, mean ± SD	−0.22	±1.21	0.14	±1.39	0.003
Age, mean years ± SD	46.8	±8.4	46.8	±8.6	-
Female sex	134	(67.7)	134	(67.7)	-
BMI, mean kg/m^2^ ± SD	22.9	±4.6	26.4	±6.4	<0.0001
Underweight <18.5 kg/m^2^	34	(16.4)	16	(7.7)	
Normal ≥18.5 and <25 kg/m^2^	113	(54.6)	82	(39.6)	<0.0001
Overweight ≥25 and <30 kg/m^2^	45	(21.7)	54	(26.1)	
Obese ≥30 kg/m^2^	15	(7.3)	55	(26.6)	
Calcium intake, mean mg/jour ± SD	457	±334	344	±296	0.0005
Current smoker	8	(3.9)	22	(10.6)	0.002
History of fracture	4	(1.9)	4	(1.9)	1
Regular physical activity	54	(26.1)	55	(26.6)	0.9
Post-menopausal	45/134	(33.6)	43/134	(32.1)	0.7
BMI at treatment initiation, mean kg/m^2^ ± SD	20.7	±3.7		-	
HCV Ab	12	(5.8)		-	
CDC C-stage	113	(54.6)		-	
ART duration, median years (IQR)	8.8	(8.0–10.0)		-	
PI				-	
Never	114	(55.1)		-	
Past use	57	(27.5)		-	
Current	36	(17.4)		-	
Duration, median months (IQR)	4.9	(3.9–6.3)		-	
TDF				-	
Never	152	(73.4)		-	
Past use	10	(4.8)		-	
Current use	45	(21.8)		-	
Duration, median months (IQR)	2.5	(1.5–2.8)		-	
Current CD4 cell count, median cells/µL (IQR)	496	(351–694)		-	
CD4 cell count nadir <100 cells/µL	81	(39.1)		-	
Current HIV viral load <50 copies/mL	174	(84.1)		-	

Note: Data are no. (%) unless otherwise indicated.

BUA, bone ultrasound attenuation; SD, standard deviation; BMI, body mass index; HCV Ab, hepatitis C virus serological status; ART, antiretroviral treatment; IQR, interquartile range; PI, protease inhibitor; TDF, tenofovir disoproxyl fumarate.

### QUS bone mineral density according to HIV infection status

**Figure 1:**
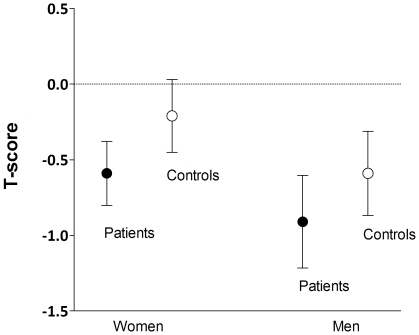
Mean QUS bone mineral density expressed as T-score (and 95% confidence interval) in HIV-infected patients and controls, separately for men and women.

The 207 patients were compared to 207 age- and sex-matched controls from the general population. HIV-infected patients had lower mean BUA and mean T-score than controls (T-score difference: −0.36±0.12; 95% confidence interval (CI), −0.59; −0.12) (Table 1). Although T-scores in men were lower than in women (−0.75±1.26 vs −0.40±1.35; P<0.0001), differences between patients and controls in the two sexes were of similar magnitude (−0.38; CI: −0.68;−0.08 and −0.32; CI: −0.71; 0.07 for women and men, respectively) (Figure 1).

Comparison between patients and controls for risk factors of low T-score indicated that BMI was much lower in patients (Table 1). Participants with a BMI <18.5 kg/m^2^ (underweight) were found twice as frequently among patients (16%) as among controls (8%). Female patients and controls had significantly higher mean BMIs than males, with considerable prevalence of overweight-obesity (37 and 67% for patient and control females, respectively, compared to 15 and 26% in men). Patients had a higher mean calcium intake per day and smoked less. History of fracture, physical activity and proportion of post-menopausal women was similar between groups.

After adjusting for factors that met the criterion of P<0.20 in bivariate analysis, i.e. BMI and physical activity, the estimated T-score difference between patients and controls was −0.25 (CI: −0.50; 0.003) and significance was borderline (P = 0.053). Additional adjustment for smoking and calcium intake led to similar results (difference: −0.27; CI: −0.53; −0.002; P = 0.048). Comparison of models including or not BMI indicated that the difference in BMI according to HIV status explained 27% of the difference in T-scores between groups.

### Factors associated with QUS bone mineral density in HIV-infected patients

In bivariate analyses, factors associated with lower T-score included older age, lower current BMI and lower BMI at treatment initiation. PI use in the past, the CD4 nadir <100 cells/µL and current viral load >50 copies/mL were also associated with a lower T-score without reaching statistical significance (Table 2). In multivariate analysis, the T-score was positively and independently associated with current BMI. BMI at treatment initiation was not included in the multivariate model because it co-varied with BMI. However, when tested separately, it was also independently associated with the T-score, although the association was weaker compared to current BMI (data not shown). The final model also suggested that undetectable viral load was associated with a higher T-score. Current or past use of PI or TDF did not show any independent association with the T-score. Additional analyses indicated that patients who had received PI were more frequently in the CDC C-stage (71.0% in the ever-received group vs 41.2% in the never-received group; P<0.001), more frequently had a CD4 cell count nadir <100 cells/µL (58.1% vs 23.7%; P<0.001) and had a lower current CD4 cell count (median: 452, IQR: [316–645] vs 536 [386–781] cells/µL; P = 0.008).

**Table 2:** Bivariate and multivariate regression analyses of factors associated with T-score in HIV-infected patients.

Characteristic		Bivariate			Multivariate	
	β^a^	95% CI	P	β^a^	95% CI	P
Age (/10 years)	−0.26	[−0.45;−0.06]	0.012	−0.19	[−0.40;0.01]	0.07
Female sex	0.32	[−0.03;0.67]	0.076	−0.04	[−0.41:0.33]	0.83
Current BMI (kg/m^2^)^b^	0.10	[0.06;0.13]	<0.001			
Underweight <18.5 kg/m^2^	Ref.			Ref.		
Normal ≥18.5 and <25 kg/m^2^	0.63	[0.18;1.08]	0.007	0.57	[0.12;1.02]	0.01
Overweight ≥25 and <30 kg/m^2^	1.23	[0.70;1.75]	<0.0001	1.19	[0.66;1.73]	<0.0001
Obese ≥30 kg/m^2^	1.19	[0.47;1.90]	0.001	1.13	[0.40;1.87]	0.003
BMI at treatment initiation kg/m^2^ c	0.08	[0.03;0.12]	0.001			
Calcium intake (/100 mg/day)	−0.014	[−0.06;0.04]	0.6			
Current smoker	−0.05	[−0.93;0.82]	0.9			
History of fracture	−0.25	[−1.48;0.98]	0.7			
Regular physical activity	0.32	[−0.06;0.70]	0.10	0.31	[−0.07;0.68]	0.11
HCV Ab	0.12	[−0.60;0.84]	0.8			
CDC C-stage	−0.13	[−0.47;0.21]	0.5			
ART duration (years)	−0.02	[−0.13;0.08]	0.7			
PI						
Never	Ref.			Ref.		
Past use	−0.35	[−0.74;0.04]	0.08	−0.09	[−0.48;0.30]	0.66
Current use	−0.30	[−0.76;0.17]	0.21	−0.18	[−0.64;0.28]	0.44
TDF						
Never	Ref.					
Past use	−0.04	[−0.84;0.75]	0.91			
Current use	−0.06	[−0.48;0.35]	0.76			
Current CD4 cell count (/100 cells/µL)	0.03	[−0.03;0.09]	0.28			
CD4 cell count nadir <100 cells/µL	−0.34	[−0.67;0.00]	0.053	−0.27	[−0.61;0.08]	0.13
Current HIV viral load <50 copies/mL	0.44	[−0.03;0.90]	0.067	0.48	[0.02;0.93]	0.04

BMI, body mass index; HCV Ab, hepatitis C virus serological status; ART, antiretroviral treatment; PI, protease inhibitor; TDF, tenofovir disoproxyl fumarate.

a β-coefficients <0 indicate an inverse association between the characteristic and T-score.

b Current BMI was kept as a categorical variable in the multivariate model because of non-linearity.

c BMI at treatment initiation was excluded from the multivariate model, as it covaried with current BMI (Pearson's correlation coefficient: 0.64).

## Discussion

Our findings indicate that Senegalese HIV-infected patients from the ANRS 1215 cohort had lower heel QUS bone mineral density than age-and sex-matched controls from the general population in Dakar. The crude T-score difference observed between patients and controls was a −0.35 standard deviation. In other studies comparing HIV-infected to uninfected individuals, the T-score difference between groups (infected/uninfected) varied from −0.25 to −1 [18], [19], [20], [21], [22], [23], [24].

We showed that the difference in QUS bone mineral density was, in part, related to the difference in BMI between patients and controls. About a third of the difference in bone mineral density was explained by a difference in corpulence. This observation is in agreement with Bolland et al., who suggested that the relationship between HIV infection and low bone density was mediated by low body weight [25]. Indeed, bone mass is known to be positively correlated with body weight or BMI, as an indicator of muscular mass, and HIV-infected individuals usually have lower body weight compared with uninfected persons [26]. Based on a meta-analysis, Bolland et al. showed that, after adjustment for body weight, residual between-group differences in bone mineral density were small (2.2–4.7%) and unlikely to be clinically significant. Similarly, a recent study conducted among veterans in the United States indicated that men with HIV infection were at increased risk of fragility fractures compared with uninfected men. However, those authors showed that this excess of risk was almost completely attributable to lower BMI among infected men [4].

Most studies comparing African-American and white American men and women have indicated higher bone mineral density in African-Americans; however, it is not clear whether this difference can be attributed to differences in BMI between the two groups [27], [28], [29]. Bone status is poorly documented in resource-limited settings like Sub-Saharan Africa. A few studies suggested that bone mineral density in Africa may be similar to northern world observations, but the prevalence of fracture appears to be much lower [30], [31], [32]. In our study, the mean Z-score observed in the control group was close to 0 (−0.08). This means that the distribution of crude BUA measurements in the control Senegalese group was fairly similar to that of reference Caucasian data provided by the manufacturer.

Most longitudinal studies involving ART-naive individuals showed that bone density declined by 2 to 6% within 24 to 48 weeks after ART initiation [33], [34], [35], [36]. Thereafter, bone mineral density values remained stable or even increased slightly [37], [38]. We did not find any association between QUS bone mineral density and duration of treatment in our study. This could be due to the fact that all patients had received at least 5 years of ART. Concerning the type of treatment, several randomized trials reported significantly greater bone loss in patients who began tenofovir-based therapy compared with other regimens [36], [39], [40]. The impact of PI on bone loss remains unclear. While some studies found that PI increased the risk of low bone mineral density [23], [35], [41], [42], others did not confirm these results [18], [20], [43]. In the present study, patients receiving or having received IP-based treatment tended to have lower T-scores compared with patients who were never treated with PI. However, PI-treated patients were also more likely to have lower nadir CD4 cell counts and lower current CD4 cell counts, and were likely to be at a more advanced stage of the disease. Multivariate analysis did not confirm an independent association between PI and T-score, indicating that PI use was more likely a marker of disease severity. Incidentally, a trend toward an association between viral load and T-score was found. Undetectable patients had higher T-scores than patients above the threshold of 50 copies per mL. The interpretation of this association is not clear. To our knowledge, only two studies had previously observed an association between high viral load and reduced bone mineral density, suggesting a potential role for the virus itself [44], [45]. In contrast, another study reported an inverse relationship; low viral load was associated with reduced bone mineral density [46].

We have shown, in an African setting, that HIV-infected patients have reduced QUS bone mineral density in comparison with subjects from the general population. However, the clinical significance of this result in terms of osteoporosis remains unknown, since we could not use the validated reference method for bone density assessment. The World Health Organization criteria for defining osteoporosis and osteopenia based on T-score is valid only for measurement of bone mineral density performed with DXA, and application of these criteria to QUS methodology is not currently recommended by the International Society of Clinical Densitometry [11]. Prospective studies of fracture risk prediction and cross-sectional equivalence studies need to be implemented in African settings to fully validate use of these alternative technologies for clinical purposes and osteoporosis management.

Finally, selection bias in the composition of both patient and control groups should be taken into account. Patients in the study cohort were those who had survived and who were able to go to the hospital for their consultation. They may have been more robust than the average patient, and therefore had higher bone mineral density. In the control group, participants were volunteers, and it can be speculated that those who agreed to take part in the study may have been healthier than those who refused to participate.

### Conclusions

We have shown for the first time, in a resource-limited setting, that HIV-infected patients on antiretroviral therapy exhibit lower bone mineral density than their uninfected counterparts. However, this reduced bone density is partly explained by lower BMI, suggesting that BMI may mediate the relationship between HIV infection and bone mineral density. Further investigation is needed to establish the clinical significance of these observations. Meanwhile, we hypothesize that starting antiretroviral therapy earlier will reduce the impact on bone by limiting the adverse effects of the infection on weight loss and nutritional status.
